# Obesity induced by a pair-fed high fat sucrose diet: methylation and expression pattern of genes related to energy homeostasis

**DOI:** 10.1186/1476-511X-9-60

**Published:** 2010-06-09

**Authors:** Almudena Lomba, Fermín I Milagro, Diego F García-Díaz, Amelia Marti, Javier Campión, J Alfredo Martínez

**Affiliations:** 1 Department of Nutrition and Food Sciences, Physiology and Toxicology, University of Navarra. Pamplona, Spain

## Abstract

**Background:**

The expression of some genes controlling energy homeostasis could be regulated by epigenetic mechanisms that may play a role in body weight regulation. Thus, it is known that various nutritional factors affect DNA methylation. In order to assess whether the macronutrient composition of the diet could be related to the epigenetic regulation of gene expression and with obesity development, we investigated the effects on methylation and expression patterns of two pair-fed isocaloric diets in rats: control (rich in starch) and HFS (rich in fat and sucrose).

**Results:**

The pair-fed HFS diet induced higher weight gain and adiposity as compared to the controls as well as liver triglyceride accumulation and oxidative stress. Feeding the HFS diet impaired glucose tolerance and serum triglycerides and cholesterol. Liver glucokinase expression, a key glycolytic gene, remained unaltered, as well as the mRNA values of fatty acid synthase and NADH dehydrogenase (ubiquinone) 1 beta subcomplex, 6 (NDUFB6) in liver and visceral adipocytes, which regulate lipogenesis and mitochondrial oxidative metabolism, respectively. Liver expression of hydroxyacyl-coenzyme A dehydrogenase (HADHB), a key gene of β-oxidation pathway, was higher in the HFS-fed animals. However, the methylation status of CpG islands in HADHB and glucokinase genes remained unchanged after feeding the HFS diet.

**Conclusions:**

These results confirm that the distribution and type of macronutrients (starch *vs*. sucrose, and percent of fat) influence obesity onset and the associated metabolic complications. HFS diets produce obesity independently of total energy intake, although apparently no epigenetic (DNA methylation) changes accompanied the modifications observed in gene expression.

## Background

Excessive weight gain arises from the interactions among environmental factors (dietary intake and physical activity), genetic predisposition and the individual behaviours [1]. Thus, a sedentary lifestyle and unhealthy diet consumption (high saturated fat and refined carbohydrates) are important determinants for the increasing prevalence of the metabolic syndrome and associated complications, namely type 2 diabetes, dyslipemia and cardiovascular diseases [2]. In fact, studies by Barnard et al. [2,3] have documented that the syndrome can be induced in rats by feeding a high-fat-sucrose (HFS) diet similar to the typical US diet. Furthermore, trials in rats have clearly shown the capacity of diets rich in simple sugars to reduce insulin sensitivity [4].

Among the different mechanisms that could lead to interindividual differences in fat deposition and obesity, the epigenetic regulation of gene expression has emerged in the last years as a potentially important contributor [1]. Exposure to nutritional, chemical, and physical factors has been postulated to influence these epigenetic events, contributing to modify the gene expression profile and potentially altering disease risks [5]. Thus, changes in DNA methylation patterns could be a result of the interplay of various dietary and environmental factors and also could be the cause of inter-individual differences concerning the susceptibility to develop obesity and other metabolic diseases [1]. In this context, not only the intake of methyl donors (betaine, choline, methionine, zinc, and folate) are likely to alter DNA methylation, but also some macronutrients such as fatty acids intake could be involved [6].

The potential reversibility of DNA methylation makes epigenetics an attractive target for therapeutic intervention [7]. The role of epigenetic factors in the regulation of gene expression makes epigenetics a major topic of interest in the onset, development and therapy of diseases such as cancer, type 2 diabetes and obesity [8]. In this context, different human genes have been described as regulated by epigenetic mechanisms in relation to metabolic diseases [1], such as HSD11B2 (hypertension), PPARG (atherosclerosis) or PPARGC1 (diabetes). Other genes involved in the development of obesity, such as leptin [9] and TNF-alpha [10], have been found to be regulated by epigenetic mechanisms influences by the diet or obesity. Other metabolic gene that is regulated by epigenetic mechanisms is NDUFB6. Thus, Ling et al. [11] demonstrated that NDUFB6 DNA methylation patterns are associated with an age-dependent decline in its expression in human skeletal muscle, opening up the possibility that epigenetic marks such as DNA methylation could predispose an individual to insulin resistance and type 2 diabetes mellitus. NADH dehydrogenase (ubiquinone) 1 beta subcomplex, 6 (NDUFB6) codifies for an inner mitochondrial membrane protein involved in mitochondrial electron transport [12].

Glucokinase (GCK) is a key gene in energy homeostasis involved in glucose phosphorylation in liver as the first step of the glycolytic pathway, and whose expression is activated in patients with type 2 diabetes [13]. Epigenetic regulation of this gene has been recently reported [14] although it has not been studied in nutritional studies. Furthermore, hydroxyacyl-coenzyme A dehydrogenase (HADHB) catalyzes two steps in mitochondrial fatty acid oxidation and its deficiency causes abnormal fatty acid metabolism [15]. Whether this gene may be regulated by epigenetic mechanisms is yet to be determined.

In summary, in order to unveil some of these questions, we have generated an animal model (HFS), whose caloric intake is similar to the control group, but varying in their macronutrient composition, since HFS diet provides more fat and sucrose and less starch than the C diet. The aim of this study was to test whether differences in dietary macronutrient composition can induce changes in the methylation patterns of several key metabolic genes, and whether these epigenetic processes could be related to changes in gene expression of these genes and on obesity development.

## Results

### Metabolic effects

By design, no differences in energy intake were found between both experimental groups. However, the animals fed a HFS diet increased their weights up in 209.2 ± 12.0 g, whereas the control group only gained 158.5 ± 7.2 g, being the differences statistically significant (p < 0.01). Moreover, neither energy expenditure nor the respiratory quotient were different between the two groups (Table 1).

**Table 1:** Food intake, respiratory quotient and energy expenditure in both experimental groups (C and HFS)

	Control	HFS	T test
Weight gain	158.50 ± 7.22	209.25 ± 12.05	**
Food intake at the end of the treatment (Kcal/d	75.1 ± 1.8	72.5 ± 2.9	NS
Respiratory quotient	0.73 ± 0.0	0.72 ± 0.0	NS
Energy expenditure (Kcal/d/bw ¾)	116.6 ± 2.8	125.7 ± 3.7	NS

NS: not statistically significant; d: day; bw: body weight. Data are given as mean ± SEM. Control vs. HFS: * p < 0.05.

The HFS diet significantly decreased total cholesterol (p < 0.01) and HDL-cholesterol (p < 0.001) levels as well as serum triglycerides (p < 0.05) as compared to the control group (Table 2). No differences in fasting glucose, FFA, lactate, insulin and adiponectin were observed.

**Table 2:** Serum and liver measurements in the C and HFS experimental groups

	Control	HFS	T test
Glucose (mg/dL)	107.5 ± 2.2	109.5 ± 3.8	NS
Cholesterol (mg/dL)	76.6 ± 4.2	59.5 ± 2.8	**
HDL-C (mg/dL)	25.6 ± 1.0	19.4 ± 0.7	***
FFA (mmol/L)	0.7 ± 0.05	0.7 ± 0.05	NS
Lactate (mg/dL)	17.8 ± 1.2	22,4 ± 2.9	NS
Serum triglycerides (mg/dL)	98.8 ± 5.9	80.7 ± 4.6	*
Insulin (μg/L)	1.0 ± 0.2	1.2 ± 0.2	NS
Adiponectin (μg/mL)	8.84 ± 2.48	8.19 ± 1.39	NS
Liver TBARS (mM TBARS/g liver)	0.95 ± 0.01	1.43 ± 0.20	*
Liver triglycerides (mmol/L)	2.53 ± 0.20	5.07 ± 0.43	***

NS: not statistically significant; TBARS: thiobarbituric acid reactive substance. Data are given as mean ± SEM. Control vs. HFS: * p < 0.05; ** p < 0.01; *** p < 0.001.

Thiobarbituric acid reactive substance (TBARS), a marker of liver lipid peroxidation, was higher (p < 0.05) in the HFS-fed group. This finding was related to an increase in liver triglycerides (Table 2), suggesting an impairment in liver metabolism as well as hepatic steatosis. In fact, a close correlation (p < 0.001) was found between both variables.

**Figure 1:**
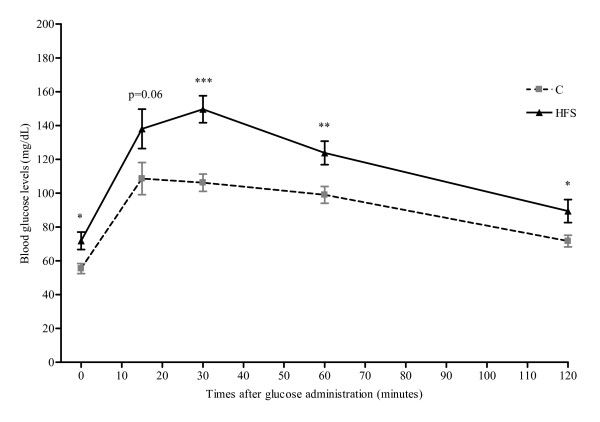
**Intraperitoneal glucose tolerance test performed in HFS and C groups**. After glucose administration (1g/Kg bw), the blood glucose levels were measured at 0, 15, 30, 60, and 120 min. Results are expressed mean ± SEM. Control *vs*. HFS: * p < 0.05; ** p < 0.01; *** p < 0.001

During the experimental trial (at week 5), when still no differences appeared in body weight between both groups, an intraperitoneal glucose tolerance test was performed. These results suggested that an incipient development of insulin resistance in the HFS animals (Figure 1).

**Figure 2:**
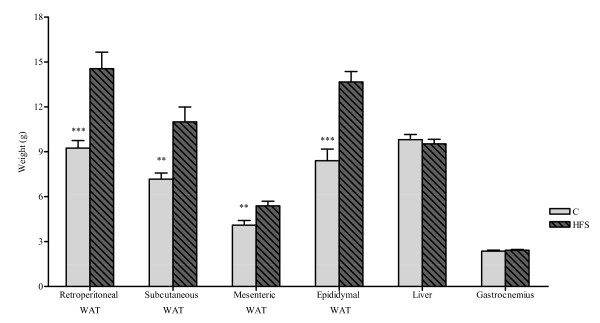
**Weight of various organs and tissues after sacrifice**. RP, retroperitoneal; SC, subcutaneous. Results are expressed as mean ± SEM. Control *vs*. HFS: ** p < 0.01; *** p < 0.001

After sacrifice, marked weight differences between both experimental groups were observed in several adipose tissue depots: subcutaneous, retroperitoneal, mesenteric and epididymal. As expected, the adiposity was higher in HFS Group as compared to C animals (Figure 2). Visceral fat was determined as the sum of retroperitoneal, mesenteric and epididymal fat and was also significantly higher (p < 0.001) in the HFS group (33.60 ± 1.86 g) respect to C (21.74 ± 1.35 g).

### Gene Expression

**Figure 3:**
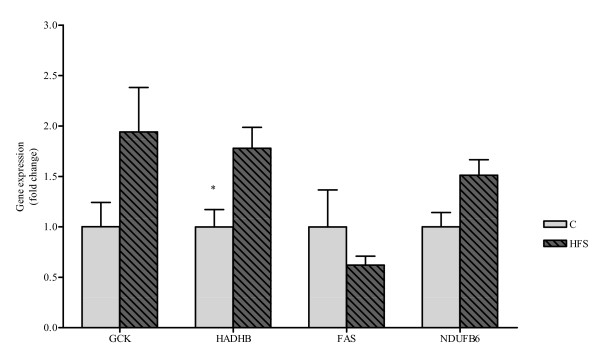
**GCK, HADHB, FAS and NDUFB6 mRNA expression**. GCK, HADHB, FAS and NDUFB6 mRNA expression values measured by RT-PCR in liver from Control and HS Groups. All results are expressed as fold of change as compared to controls (control set at unity), and shown as mean ± SEM. Control vs. HFS: * p < 0.05

Additionally, the liver mRNA levels of four genes were analyzed: HADHB, GCK, FAS and NDUFB6. Interestingly, the HADHB expression was statistically higher (p < 0.05) in the group fed a HFS diet as compared to C group. No statistical differences were found in the other three genes, GCK, FAS and NDUFB6, despite a slight increase in GCK expression (Figure 3). The mRNA levels of FAS, NDUFB6 and HADHB were also analyzed in epididymal adipose tissue, although no differences were observed between both dietary group (data not shown).

### DNA Methylation

**Figure 4:**
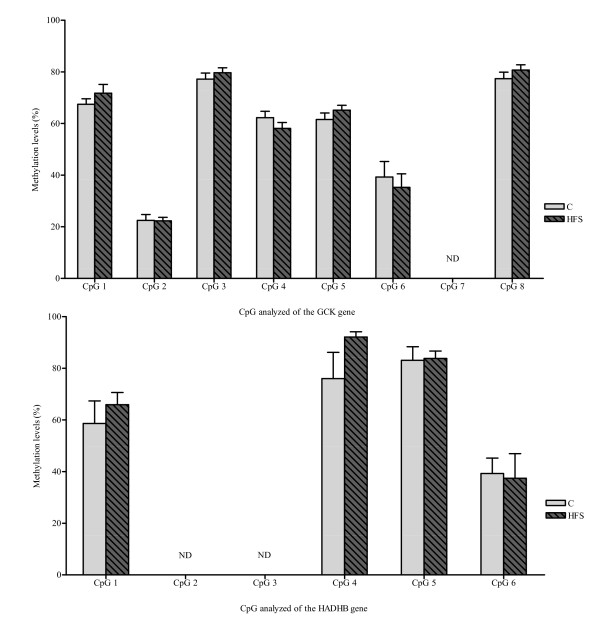
**Methylation percentages of GCK and HADHB genes**. Methylation percentages obtained for each of the CpGs analyzed for GCK (above) and HADHB (below) in liver. There were no statistically significant differences induced by the dietary groups. In GCK gene, CpG 7 levels were not identified in the analysis. CpG 2 and CpG 3 were not identified in HADHB gene analysis. Not detected, ND. Results are expressed mean ± SEM

When analysing the methylation pattern of GCK in liver and HADHB in liver and epididymal fat, no relevant differences were found between the dietary groups (Figure 4).

## Discussion

The current trial asserts the important role of the dietary macronutrient distribution on weight gain. Indeed, the amount and composition of the ingested food appeared to influence body-weight regulation [16]. Thus, the HFS group showed a statistically significant increase in body weight at the end of the dietary treatment compared with the C group, in spite of isocaloric intake. Other studies have reported comparable results in rodents fed *ad libitum *with a similar diet [3], but apparently never within a *pair-fed *model. In fact, a pair-feeding model of HFS diet was performed in Sprague-Dawley rats, although no changes in body weight gain were observed [17]. The strongest evidence of the experimental hypothesis comes from rodent studies in which a high-fat (HF) diet induced obesity independently of total energy intake [18,19]. This kind of experiments is very difficult to perform in humans.

Our results confirm that the dietary distribution and the type of macronutrients (starch *vs*. sucrose and unsaturated *vs*. saturated fats) could influence the development of metabolic disorders. Thus, when the intraperitoneal glucose overload was performed, the curve of glucose tolerance was higher and prolonged in time in the HFS group, as described by other authors after consuming *ad libitum *HFS [20], HF [21] and high sucrose diets [22]. In this context, in *ad libitum *dietary models, insulin resistance appeared after 2 weeks, without important changes in plasma triglycerides, plasma glycerol, or blood pressure, even before any indication of obesity or adipocyte hypertrophy [2]. Thus, Grimditch et al. [23] previously reported that, of the two main components of the HFS diet, refined sugar caused a greater problem than the high fat content during glucose tolerance tests. However, the combination of high fat and high refined sugar resulted in an ever worse response.

On the other hand, Barnard et al. [2] have demonstrated that the insulin resistance onset occurs before other metabolic syndrome manifestations, being the dietary intake, but not excessive weight gain, the underlying cause. In addition, studies in humans have also indicated that insulin resistance usually precedes other aspects of the metabolic syndrome such as dyslipidemia and hypertension [24]. In this context, it is likely that at the time of intraperitoneal glucose overload, the HFS group was developing a preliminary state of insulin resistance, characterized by a lower insulin sensitivity unable to compensate glucose overloads. However, in the fasting condition after sacrifice, no differences in serum insulin levels were observed.

Total and HDL-cholesterol circulating levels significantly decreased in the HFS group dietary treatment. This reduction could be, in part, the result of the lower HDL cholesterol levels, as has been previously reported by Boqué et al. [25] using a similar diet (*ad libitum*) in Wistar rats, although these differences were not statistically significant. It is known that the type of dietary carbohydrate (starch versus sucrose) [26] and the type of fat in the diet [27] affect cholesterol metabolism in rats. Likewise, the serum triglyceride levels were significantly lower in the HFS group as compared to C, as other authors have previously described with a HFS diet. Thus, Boqué et al. [25] reported a similar outcome in Wistar rats fed a HFS diet *ad libitum *for 35 days, Yamamoto et al. [28] in Sprague-Dawley rats fed a HFS diet during 4 weeks, and Jourdan et al. [29] in mice fed for more than 20 weeks. These results suggest an increase in TG clearance by the liver and adipose tissue, and confirm the lipid metabolism differences between rodent and human models.

In relation to hepatic TG, it has been reported that the consumption of one-third more calories in the form of dietary fat but not carbohydrates produces steatosis in rats [30]. These authors indicate that the delivery and utilization of this extra fat by the liver in the HF *ad libitum *group provides the first hit in the development of Non-Alcoholic Fatty Liver Disease (NAFLD) in Sprague-Dawley rats.

In this context, TBARS is considered a reliable marker of cellular lipid peroxidation [31]. Our results demonstrate that HFS intake induces an increase in liver TBARS in comparison with the animals fed on the C group. Other authors have reported similar increases in oxidative stress with *ad libitum *HFS [28], HF [32] and cafeteria diets [33]. Lipid peroxidation leads to reduced hepatic mitochondrial oxidative capacity and increased susceptibility to hepatic steatosis and injury [34]. In this sense, saturated fatty acids may promote endoplasmic reticulum stress and hepatocyte injury resulting in hepatic dysfunction in rodents [35]. The inflammatory status related to obesity may be also originated by oxidative stress, which induces cell injury and could be able to dysregulate adipocytokine production and insulin sensitivity [36].

Concerning mRNA studies in liver, HADHB gene expression was significantly increased in HFS animals as compared to C. This gene encodes an inner mitochondrial membrane protein made up of α and β subunits that contributes to three of the four reactions necessary for mitochondrial long-chain fatty acid β-oxidation [37]. The β-subunit shows 3-oxoacyl-CoA thiolase activity, and the α-subunit provides enoyl-CoA hydratase and 3-hydroxyacyl-CoA dehydrogenase activity. Concerning dietary regulation of this gene, Oshida et al. [38] reported that HADHB activity in muscle was higher in rats fed a high sucrose diet. According to them, the high sucrose group exhibited a metabolic pattern in muscle that favoured carbohydrate over fat oxidation. A later study in humans demonstrated that HADHB activity increased when the palmitate oxidation occurred [39]. These results suggest that this enzyme performance is increased in the presence of fatty acids.

Regarding to GCK gene expression, we observed a slight increase (although not statistically significant) in the HFS group, similarly to others authors [40]. Glucokinase catalyses the first and rate-limiting step in glycolysis: the phosphorylation of glucose to glucose-6-phosphate. In the liver, GCK activity determines the rate of glucose utilization and glycogen synthesis [41]. In rats, diets enriched in fat or sucrose are able to: 1) reduce the ability of insulin to suppress glucose production in vivo, 2) decrease the ability of insulin to suppress gluconeogenesis in liver, and 3) increase the capacity for gluconeogenesis from a number of precursors in liver [42,43]. The importance of GCK in humans is evidenced by the fact that heterozygous loss-of-function mutations cause maturity-onset diabetes of the young type 2 (MODY 2), a disease characterized by early-onset and persistent hyperglycaemia [44].

With regard to epigenetic studies, no changes in HADHB and GCK methylation patterns were observed. Apparently, we are the first to investigate the possible HADHB epigenetic regulation, while, in relation to GCK, only one publication has addressed the study of the methylation of this gene, although in relation to the aging process [14].

In this context, Ling C et al. [11] demonstrated that NDUFB6 DNA methylation played a role in the pathogenesis of insulin resistance and type 2 diabetes mellitus. However, we have not found differences in relation to dietary macronutrient changes.

## Conclusions

The main conclusion of this work is that the different macronutrient distribution of the diet, with no differences in terms of total energy intake, may play an important role in weight gain and metabolic syndrome manifestations, by affecting the expression of key energy metabolism genes. However, the methylation patterns in the investigated gene regions remained apparently unaffected, suggesting that gene expression changes are not likely to be due to modifications in DNA methylation.

## Methods

### Animal and Diets

Eight-weeks-old male Wistar rats (initial weight 254g ± 4; n = 24), supplied by the Applied Pharmacobiology Center (CIFA, Pamplona, Spain), were individually housed at 21-23°C and controlled (50 ± 10%) humidity under a 12 h artificial light cycle (8 a.m. to 8 p.m.) The animals were randomly assigned to two different dietary groups: Control and High Fat Sucrose (HFS). The control animals (n = 12) were fed a standard pelleted diet (2014 Tekland Global 14% Protein Rodent Maintenance Diet, Harlan Iberica, Barcelona, Spain) containing 20% of energy as proteins, 67% of energy as carbohydrates (5% sucrose, 62% starch) and 13% of energy as fat by dry weight, while the HFS group (n = 12) was fed a high fat sucrose diet (D12451, Research Diets, New Brunswick, NJ, USA). The composition of this diet was 20% of energy as protein, 35% as carbohydrates (18% sucrose, 10% maltodextrin and 7% starch) and 45% as lipids by dry weight.

During the experimental trial, the HFS group received a diet restricted to the amount of calories (kcal / [g body weight]) that the C group had consumed the day before (*pair-fed *model), as previously described [19]. Body weight and food intake were daily recorded.

### In vivo experiments

At week 5, two gas exchange determinations (oxygen [O_2_] consumption and carbon dioxide [CO_2_]) were performed by using an Oxylet 00 O_2_/CO_2 _indirect calorimeter (Panlab SL, Barcelona, Spain) as previously described [45]. Also, after a fasting period of 12 h, rats fed the HFS and C diets were intraperitoneally injected with glucose at 1g/kg body weight [46]. We collected blood from tail veins at 0, 15, 30, 60, and 120 min after glucose administration in order to determine blood glucose levels with a Glucosemeter device (Roche Diagnostic, Mannheim, Germany).

At the end of the experimental period (69 days), rats were sacrificed by decapitation. Blood and tissue samples were immediately collected and stored at -80°C for further analyses. All the procedures were performed according to national and institutional guidelines of the Animal Care and Use Committee at the University of Navarra.

### Serum and Liver analysis

Circulating total and HDL-cholesterol were measured with Cholesterol CP and HDL direct CP (ABX diagnostic, Montpellier, France) respectively in a COBAS MIRA equipment (Rochel, Basel, Switzerland). Serum triglycerides and lactate were measured with a Randox kit and a L-Lactate kit, respectively (Randox, LTD Laboratories, Ardmore Road, UK). FFA (free fatty acids) were quantified with a NEFA C kit (Wako Chemicals, Neuss, Germany). Lipid peroxidation was measured as thiobarbituric acid reactive substance (TBARS assay Kit, Cayman Chemical Company, USA) after sonicating for 15 seconds at 40 V in RIPA buffer with protease inhibitors and centrifugating at 1.600 × g for 10 minutes at 4°C. Liver triglycerides levels were determined by a colorimetric assay (Triglycerides MR, Cromatest; Linear Chemicals, Spain) after 15 seconds sonication at 40 V in Tris-Cl buffer. Serum adiponectin (Linco Research, St Charles, MO, USA) and insulin levels (Mercodia AB, Uppsala, Sweden) were measured by ELISA in an automatized TRITURUS equipment (Grifols International S.A., Barcelona, Spain).

### Gene Expression analysis

Total RNA was isolated from epididymal adipose tissue and liver according to Tri^® ^manufacturer's instructions (Sigma-Aldrich, Missouri, USA). DNase treatment was performed with a DNA-free™ kit (Applied Biosystems, Austin, TX, USA), and cDNA synthesized using M-MLV reverse transcriptase (Invitrogen, Carlsbad, CA, USA) as described by the suppliers. Quantitative Real-Time PCR assays were performed following manufacturer's recommendations using an ABI PRISM 7000 HT Sequence Detection System and Taqman probes for rat HADHB (Rn00592435_m1), GCK (Rn00688285_m1), FAS (Rn01463550_m1) and NDUFB6 (Rn03416136_m1). The gene expression levels were normalized with ubiquitin (Rn01789812_g1) and GAPDH (Rn 99999916_s1) mRNAs as internal controls (Applied Biosystems, Austin, TX, USA) and using the Genorm software [47]. Fold change between HFS and control rats was calculated using the 2^-ΔΔCt ^method.

### DNA Methylation analysis

The quantitative analysis of GCK and HADHB degree of DNA methylation was determined by using the MassARRAY system (San Diego, CA, USA), which combines base specific enzymatic cleavage with MALDITOF Sequenom mass spectrometry technology [48], after bisulphite treatment (Epitect kit, Qiagen, Valencia, CA, USA). Primers used are shown in Table 3.

**Table 3:** Primer sequences for quantitative analysis of the degree of DNA methylation using the MassARRAY system

Gene	Forward primer (5'→ 3')	Reverse primer (3'→ 5')
GCK	TAGAATTAGGGAGGAAGTAGGAAGG	GGATTTTGAGGTATTGTATTGATTTTT
HADHB	TTTTTTTATTAGTAGGAAAAGGGTAGT	GGGTTGATTTTTGAATAAGATTTGA

The following two sequences were analyzed: GCK gene, with 8 CpGs, and HADHB, with 6CpGs (Table 4). They are numbered 1-8 and 1-6 respectively in the text and figures.

**Table 4:** Methylation patterns analyzed in GCK and HADHB genes

Gene	Analyzed secuence
GCK	GTTAAGGTTAGGAGTTGTTGAGTAATTTTTAAGGTTTTTTTAGAGGTTTTGGTAGATGA
	TATTATTAGTAAAGGGTTTAATTTATGTAGTATTAATATGTTTT**C**^1^**G**GGTGAATAGATTT
	GTATGAGTTTAATGTATATTTATTTTTTAATGGAATTATTTAGGAAGTTAAAGGTTTATT
	TTGTTTTT**C**^2^**G**TTTTATTTAGAAAAAAAAAAAAAAAAAAAAAATTTTAAATTGGTGGTT
	TTAGGTGAAAGT**C**^3^**G**AGTTTTTTTTGAAATTTTGTTGTTTTTTGGTAATTTTTATGT**C**^4^**G**T
	ATTGTTTTTGAT**C**^5^**G**GTTTTATTTTTTTGAATAAATATAAGTATTGGTATTTATAGGGTTT
	AA**C**^6^**G**TTTATATTTGTATATTTA**C**^7^**G**TATTGGAGTAGATTTTTAGTATAATGTTTTTTAGT
	TTTTTTTATAGAT**C**^8^**G**
HADHB	TTTTGTAATTATTATAGTTAGTTGTGAAATTGTTAGGAA**C**^1^**G**TTTATAATAGTGTGTTT
	TTTTTAGTTTTTTTTTTTTTTTTTTTTTTTTTTT**C**^2^**G**GGGTTGGGGAT**C**^3^**G**AATTTAGGAT
	TTTG**C**^4^**G**TTTTTTAGGTAAG**C**^5^**G**TTTTATTATTGAGTTAAATTTTTAATTTTTTTTTTTAG
	TTTTTAATTGAAGTAAATTGTTTTTATTGGAGATGTTTTTGTTTGTTAGGGTTGATGTAA
	TATATAAGAATATATTTTATTTTGGTTATTATAAATTTTTTTAAATTTTTGATAATTGTG
	TTATATAATTATATTTATGTTGTGAGAGTTGTAGTTTTAGTTATAGATATTTGTTTGAAT
	GTTGTTTAAATGTATGGAAGTTGTTATTAGAATTATTTTGGTTATT**C**^6^**G**AGTATTTTTAG
	GTAAGGAATA

### Statistic analysis

Results are presented as means ± SEM of the means. Statistical analyses were performed using SPSS 15.0 packages of Windows (Chicago, IL, USA). Data were analysed by the student-T test. The Pearson correlation coefficient was used to assess the relationship between liver TBARS and triglyceride levels. Statistical significance was set-up at the P < 0.05 level.

## Competing interests

The authors declare that they have no competing interests.

## Authors' contributions

AL performed most of the experimental trial. AL, FIM, JC, and JAM developed the study together and designed the experiment. AL and DFG performed the in vivo measurements. AL, FIM, AM and JAM obtained grant funding and wrote the manuscript. All authors read and approved the final manuscript.
